# Exergaming in Older Adults: Movement Characteristics While Playing Stepping Games

**DOI:** 10.3389/fpsyg.2016.00964

**Published:** 2016-06-24

**Authors:** Nina Skjæret-Maroni, Elise K. Vonstad, Espen A. F. Ihlen, Xiang-Chun Tan, Jorunn L. Helbostad, Beatrix Vereijken

**Affiliations:** ^1^Department of Neuroscience, Faculty of Medicine, Norwegian University of Science and TechnologyTrondheim, Norway; ^2^Research Department, Sunnaas Rehabilitation HospitalNesodden, Norway; ^3^Department of Clinical Services, St. Olav’s University HospitalTrondheim, Norway

**Keywords:** aged, stepping, exergames, movement characteristics, movement analysis, motion capture

## Abstract

Despite frequent use of exergames in intervention studies to improve physical function in older adults, we lack knowledge about the movements performed during exergaming. This causes difficulties for interpreting results of intervention studies and drawing conclusions about the efficacy of exergames to exercise specific functions important for the elderly population. The aim of the current study was to investigate whether game and game level affect older adults’ stepping and upper body movements while playing stepping exergames. A 3D-motion capture experiment was performed with 20 elderly (12 women and 8 men; age range 65–90 years), playing two exergames, The Mole from SilverFit and LightRace in YourShape: Fitness Evolved, on two difficulty levels, with five 1-min trials for each game and level. Reflective markers were placed on bases of first toe, heels, and lower back. Movement characteristics were analyzed with a linear mixed model. Results indicated that both game and game level affected movement characteristics. Participants took shorter steps and had lower step velocity when playing The Mole compared to LightRace, while The Mole prompted more variation in step length and step velocity. Compared to LightRace, The Mole elicited larger upper body movements in both ML- and AP-directions and participants’ feet and upper body covered a larger area. Increasing difficulty level from Easy to Medium resulted in overall decrease of movement, except for number of steps and step speed when playing LightRace. Even with only two games, two levels, and five trials at each, this study indicates that the choice of exergame is not indifferent when aiming to exercise specific functions in older adults and that exergames need to be chosen and designed carefully based on the goals of the intervention.

## Introduction

The European population is rapidly aging and we have a longer life expectancy than ever before. With advancing age comes increased risk for disease and functional decline, which again may increase the risk of cognitive impairment, frailty, and falls, with concomitant negative consequences for quality of life (Horak et al., 1989; Bolandzadeh et al., 2015; Bridenbaugh and Kressig, 2015). Falls in particular can have dramatic consequences in terms of fractures and fear of future falls, and are the leading cause for loss of independence among older adults (Hausdorff et al., 2001; Kannus et al., 2005). In order to keep the additional years added to life expectancy mostly healthy years, maintaining regular physical activity is heralded as one of the most important lifestyle factors (Chodzko-Zajko et al., 2009).

Studies have shown that physical activity is one of the most important pathways to improve and maintain health in older adults (Chodzko-Zajko et al., 2009; Montero-Fernandez and Serra-Rexach, 2013). For example, exercise interventions among older adults living in the community can reduce falls by 17–30% (Sherrington et al., 2011; Gillespie et al., 2012). A considerable proportion of falls can be attributed to incorrect weight-shifting activities, such as during walking (Topper et al., 1993; van Dieën and Pijnappels, 2008; Robinovitch et al., 2013) or in situations that require performance of several tasks simultaneously (Melzer et al., 2010). In situations like these, older adults are more likely than younger counterparts to make steps that are too slow, in the wrong direction or too short, or to collide one leg against the other during compensatory crossover steps (Maki and McIlroy, 2006; Hsiao-Wecksler and Robinovitch, 2007).

One of the most important components in exercise interventions aimed at reducing falls among older adults is balance training (Sherrington et al., 2008). In many intervention studies, balance training consists of controlling the center of mass over a reduced base of support (Sherrington et al., 2008). However, daily life situations such as a slip, trip, or trying to avoid an obstacle during walking, require fast and corrective stepping movements that involve changing the base of support relative to the center of mass in order to correct or prevent loss of balance. The abilities to initiate a fast voluntary step or to inhibit a preplanned step and find an alternative foot landing position to avoid instability, have been found to deteriorate with age (Lord and Fitzpatrick, 2001; Potocanac et al., 2014, 2015). Additional changes in gait with increased age include lower gait speed, shorter step length, and a wider base of support, longer double support, as well as increased variability in step time, step length and step-width (Himann et al., 1988; Winter et al., 1990; Hausdorff et al., 2001).

Exercise interventions with focus on performing appropriate, rapid, timed, and well-directed steps have a valuable role in interventions and rehabilitation for older adults, and it has been found that both reactive and volitional stepping interventions reduce falls among older adults by approximately 50% (Okubo et al., 2016). Thus, exercise interventions for older adults should not focus on standing balance tasks alone, but should also include activities that focus on improving stepping, with varying speeds, multi-directional weight-shifts, dual tasking, and gaze shifting (Patla et al., 1992; Lundin-Olsson et al., 1997; Gillespie et al., 2012; Hackney and McKee, 2014; Balasubramanian, 2015).

New technology-based exercise methods, such as exergames that open up for full-body interaction with computer games, appear promising as a means to promote and maintain physical activity. Step-based games such as the Dance Dance Revolution (DDR) drew considerable attention in the early stages of exergaming research in older adults, much due to the low cost and the interaction between the sensory, information-processing, and neuromuscular systems during controlled body weight transfers (de Bruin et al., 2010). With increasingly advanced technology, exergames now have the potential to stimulate more complex and dynamic movements that contain variations in step length, direction, and speed, as well as inhibition of voluntary step initiations and avoidance of virtual obstacles. These are all skills that are necessary to successfully navigate through different situations in daily life.

A rapidly growing number of studies comparing exergame-based exercise interventions with other interventions have found that exergames show either comparable or slightly better changes in physical functions (e.g., Laver et al., 2012; Cho and Lee, 2013; Duque et al., 2013). However, we lack knowledge about the specific movements that players perform during exergaming, making it difficult to interpret results of intervention studies and to draw conclusions about the efficacy of exergames to exercise and improve specific functions important for the elderly population. The limited research that has been conducted on movement quality and movement patterns in commercial exergaming, have been on younger gamers (Pasch et al., 2009), healthy children (Levac et al., 2010), and children with cerebral palsy (Berry et al., 2012). These studies suggest that movements performed during gaming are highly variable between players and that it might be necessary to explore more games in order to ensure that the intended movement characteristics are practiced during game play (Pasch et al., 2009; Berry et al., 2012). Furthermore, it is unclear to what extent players can do meager renditions of the intended movements that yield scoring of points despite these renditions not being the specific movement characteristics that are required to achieve the intended exercise (Pasch et al., 2009; Levac et al., 2010). In addition, increasing game level is a natural part, and often a goal, in gaming. However, it is unknown how increasing difficulty level, for example by adding a cognitive element, having additional movements in the game, or increasing game speed, affects the movement characteristics older adults display during gaming.

There are promising indications that stepping exergames may be applicable to improve physical and cognitive functions that are necessary to successfully navigate through different situations in daily life. However, to date, no studies have been published that focused on the movement characteristics people display when playing stepping exergames. We lack knowledge about whether stepping games actually result in players moving their entire body while taking steps, whether they take large or small steps, and whether they vary their steps during a game session. The current study is the first to address these gaps in our knowledge by objectively investigating the movement characteristics that older adults display when playing step-based exergames. Two different stepping exergames were chosen, based on a previous observational study investigating the relationship between relevant aspects of stepping behavior during game play and game elements, where The Mole and LightRace elicited better overall movement quality compared to DDR (Skjaeret et al., 2015). The main aim of the current study is to investigate whether differences in game, difficulty level, and short-term experience affect movement characteristics of the players during game play, by describing step and upper body characteristics displayed by older adults.

## Materials and Methods

### Participants

A convenience sample of 20 community-dwelling older adults (12 female, 8 male) participated in the study. To be included, participants had to be over the age of 65 years, have no known physical or mental disabilities, and be able to walk safely without a walking aid. The participants were recruited from recreational exercise groups in the municipality of Trondheim, Norway. All subjects provided informed, written consent. The study was approved by the Regional Ethical Committee for Medical and Health Research Ethics, and conducted in accordance with the Declaration of Helsinki.

### Exergames

**FIGURE 1:**
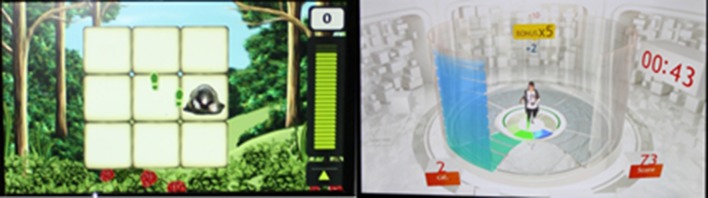
**Screen captures from game play of The Mole from SilverFit (left) and LightRace in YourShape: Fitness Evolved (right).** The left panel shows the easy level of The Mole, with the green feet representing the player, and the target, a mole, in the middle right square. The right panel shows game play of LightRace where the player steps on the front (left) panel, the correct target, turning the specified area on the screen green.

Based on a previous observational study on older adults’ movement characteristics during stepping exergames (Skjaeret et al., 2015), two step-based exergames were chosen for the current study, The Mole from SilverFit (SilverFit BV, Woerden, The Netherlands), and LightRace in YourShape: Fitness Evolved (Ubi Soft Divertissement Inc., Montréal, QC, Canada) (see **Figure 1**). Both games require the players to take steps in different directions and hit a target in order to play the game and score points.

SilverFit is a virtual reality rehabilitation system with several mini games specifically designed for older adults in exercise and rehabilitation settings (Rademaker et al., 2009). The system uses a 3D motion-sensing camera to detect the player’s movements during gaming, and shows the displacement of the player’s feet on the screen. In the current study, the mini game The Mole was played at two levels of difficulty, “Basic” and “Precision Control.” The game is played by stepping on stationary moles and moving mice to chase them from a garden (Basic) while avoiding stepping on lady bugs (Precision Control). When stepping on the correct target the square turns green and an affirmative sound is played. All animals appear randomly on the screen, prompting the player to move in all directions.

LightRace in YourShape: Fitness Evolved is one of a variety of mini games designed for the general public on Xbox One (Microsoft Inc.), using a Kinect motion-sensing camera to detect the player’s movements during gaming and visualize these on the screen in the form of a full-body avatar. The player stands in front of the screen and has to step on the area that lights up around the avatar on the screen. At the Easy level, lights appear in front or to the side of the player, while at the Medium level lights appear behind the player in addition. Stepping on the correct area turns the specified area on the screen green, an affirmative sound is played, and a blue path shoots up.

### Experimental Setup

An Oqus Motion Capture System (Qualisys AB, Gothenburg, Sweden) was used to track the movements of the participant’s feet and upper body. Five passive reflective markers were attached with double-sided tape bilaterally to the participant’s base of first toe, mid calcaneus, and approximately at the third lumbar vertebrae of the back. Seven infrared cameras were placed around the area where the exergames were played, and a digital video recorder was placed on the participants’ right side. As both Oqus and Kinect cameras use infrared light, they could not be placed in each other’s line of sight, as this would cause interference between the cameras. Therefore, the Oqus cameras could not be placed directly behind the participants. The Oqus cameras were calibrated to measure marker position to within 1.0 mm accuracy at a frequency of 100 Hz.

### Procedure

All participants played each exergame on two difficulty levels, Easy and Medium, with five 1-min trials for each game and level, giving a total of 20 trials for each participant. Each exergame was demonstrated to the participant and each participant received one try-out trial for each game and level before playing the five recorded trials. There was a 1-min break after each trial, and a 5-min seated break between levels and games. The games were played in counter-balanced order across participants, and they always started with the easy level of the game. One researcher stood near the participant to ensure safety while playing the exergame. After each game level, the participants marked the BORG Perceived Exertion Scale (score 6–20) (Borg, 1998) and answered a few short questions about enjoyment, possible usage, and fear of falling while playing. After completing both levels of each game, the participants filled out the Norwegian version of the Systems Usability Scale (SUS) questionnaire (score 1–100) (Brooke, 1996) to evaluate the usability of each system.

After finalizing both games, participants filled out the Norwegian version of the 16-item Falls Efficacy Scale – International (FES-I) questionnaire that measures the level of concern for falling on a 4-point scale (score 16–64) (Yardley et al., 2005; Helbostad et al., 2010). In addition, they filled out a background questionnaire regarding age, gaming experience, medication, and weekly amount of physical activity. The latter was calculated based on answers given in the questionnaire about frequency and duration of physical activity during an average week (Kurtze et al., 2008). Finally, two functional tests were performed: a 6 m walk test (Guralnik et al., 1994) and a 30 s sit-to-stand test (Rikli and Jones, 1999).

The start and end points for the walk test were marked on the floor with tape at a total distance of 10 m. The two first and last meters, i.e., the acceleration and deceleration phases, respectively, were not included in the calculation of gait speed. Participants were instructed to walk as fast and safely as they could, and gait speed was measured with a stopwatch.

For the 30 s sit-to-stand test a straight-backed armless chair with a seat height of 47 cm was used. The chair was stabilized by placing it against a wall. Participants were instructed to sit in the middle of the chair, feet flat on the floor with arms folded across the chest and rise to a full standing position and then sit back again as many times as possible in a 30-s time period.

### Data Processing and Analyzing

A custom-made Matlab script (Mathworks, Natick, MA, USA) was used to identify step characteristics and upper body movements. Step initiation and termination was identified by velocity of the toe markers, with a cut-off at ±0.1 m/s, as the heel markers were missing in several data sets. A step was set as a ≥0.03 m displacement of the toe marker lasting for ≥0.05 s. Spurious steps were eliminated after visual check of all records (less than 2%). The following parameters were calculated from the identified steps: number of steps per minute and mean and SD of step length and step velocity for both feet combined. Upper body movements were registered by the marker placed at the lower back, and parameters were calculated separately for medio-lateral (ML) and anterior-posterior (AP) directions. The following parameters were calculated for upper body movements: mean and SD of upper body velocity in ML and AP directions. Area covered by the feet and the upper body markers was calculated as the area of an ellipsoid with the length of the principle axis set equal to 1.96*SD of the variation identified by singular value decomposition (Oliveira et al., 1996). In addition, a movement area ratio was calculated as 
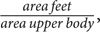
 where values close to 1.0 indicate that the movements of the feet and the upper body cover equal-sized areas, whereas larger values indicate that the feet cover a larger area than the upper body.

### Statistics

Potential redundancy of the independent variables was checked with Spearman’s correlations. Correlations between the 11 movement characteristics varied from 0.014 to 0.883, indicating that none of the independent variables were redundant. Independent samples *t*-tests were used to check whether independent variables differed significantly between fallers and non-fallers. A linear mixed model for repeated measures was used to analyze characteristics of step and upper body movements with fixed effects of Game (2), Level (2), and Trial (5), and interactions between them. The same analysis with Trial as a covariate did not affect the results. The restricted maximum likelihood method (REML) was used for the estimation of fixed and random effect parameters. All statistical analyses were performed with IBM SPSS Statistics version 22. Statistical level of significance was set at *p* < 0.05.

## Results

### Participants and Background Information

Participant characteristics are shown in **Table 1**. All participants were retired, independently living elderly. Three of the participants had some previous experience with game consoles designed for entertainment, but none of them had used exergames on a regular basis. During game play, all participants expressed high levels of enjoyment when playing both exergames, and participants preferred the Medium level to the Easy level due to the increased cognitive and physical challenge.

**Table 1:** Participant characteristics.

	Mean (*SD*)	Range
Age (years)	75.7 (5.4)	65–90
Height (cm)	167.6 (10.5)	152–185
Weight (kg)	74.6 (9.8)	54–91.5
Daily prescription medication (n)	2.0 (1.7) (median 1.5)	0–7
FES-I (score)	19.5 (2.8)	16–27
Experienced a fall within the last year (n, %)	6 (30%)	
Walking speed (m/s)	1.4 (0.3)	0.5–1.8
30 s Sit-to-stand (n)	14.6 (2.9)	8–19
Physical activity (min pr. day)	61.0 (27.7)	26.8–150

None of the falls had resulted in an injury. Independent samples *t*-tests on all independent variables indicated that there were no significant differences between those who had reported one or more falls the previous year and the non-fallers. The BORG-scores ranged from 6 to 15 for both games and levels, indicating low to medium perceived exertion. Both exergames were given above average scores (>70 on a scale from 1 to 100) on the System Usability Scale (SUS), with The Mole having a slightly higher average SUS-score (80.7 ± 15.2) than LightRace (75.8 ± 19.2).

### Game Score

In both exergames a game score was calculated and displayed for the participants while playing. The participants scored significantly more points at the Easy levels than at the Medium levels in both The Mole (30.5 vs. 23.1, *p* < 0.001) and LightRace (114.3 vs. 84.8, *p* < 0.001), giving a relative difference in score between the levels of 24.3% and 25.8%, respectively. While there was no significant difference between the levels in The Mole (*p* = 0.280), participants achieved a significantly higher game score at the Easy level compared to the Medium level when playing LightRace (29.48, CI 95% 15.43 – 43.53, *p* < 0.001). There was also a significant difference between the two games at both Easy (68.37, CI 95% 48.57 – 88.36, *p* < 0.001) and Medium levels (46.7, CI 95% 26.77 – 66.72, *p* < 0.001), reflecting that the games had different scoring systems. In addition, there was a significant interaction between Game and Trial [*F*(1,374.02) = 4.59, *p* = 0.033], indicating that the game score increased across trials when playing LightRace, while there was no such increase in the score for The Mole.

### Movement Characteristics

In general, the two exergames and the two difficulty levels within each game elicited different step and upper body movements in the participants during gameplay. **Table 2** shows the mean and standard deviation for all 11 movement characteristics and the calculated movement area ratio for each game and level across all participants and all trials. Also indicated are the significant differences between the games and levels from the linear mixed model. More detailed description of the results and statistical tests from each characteristic are provided in the following sections.

**Table 2:** Group means (SD) for all movement characteristics and the calculated movement area ratio for The Mole and LightRace at Easy and Medium levels.

	The Mole Easy	The Mole Medium	LightRace Easy	LightRace Medium
Number of steps per minute^a,b,c,d^	97.85 (3.86)	86.25 (3.68)	86.51 (4.42)	104.14 (5.04)
Step length (cm)^a,b,c,d^	34.45 (0.96)	30.57 (0.98)	42.52 (1.67)	39.4 (1.2)
SD step length (cm)^a,c^	18.06 (0.84)	16.2 (0.61)	16.34 (0.69)	16.31 (0.62)
Step velocity (m/sec)^a,b,d^	11.32 (0.27)	10.83 (0.35)	11.58 (0.38)	12.28 (0.33)
SD step velocity (m/sec)^b,c,d^	4.03 (0.19)	3.92 (0.16)	3.4 (0.16)	3.71 (0.18)
Upper body velocity				
AP-direction (m/sec)^a,b,c,d^	2.36 (0.09)	1.8 (0.09)	1.24 (0.09)	1.05 (0.08)
Variation in upper body velocity				
AP-direction (m/sec)^a,b,c,d^	2.05 (0.08)	1.73 (0.08)	1.32 (0.08)	1.1 (0.08)
Upper body velocity				
ML-direction (m/sec)^a,b,c,d^	1.99 (0.04)	1.7 (0.06)	1.5 (0.1)	1.31 (0.09)
Variation in upper body velocity				
ML-direction (m/sec)^a,b,c,d^	2.02 (0.07)	1.81 (0.07)	1.42 (0.11)	1.23 (0.1)
Area upper body (m^2^)^a,b,c,d^	0.54 (0.06)	0.5 (0.05)	0.15 (0.04)	0.09 (0.02)
Area Feet (m^2^)^a,b,c,d^	0.73 (0.05)	0.66 (0.05)	0.42 (0.03)	0.38 (0.02)
Movement area ratio^b,c,d^	1.57 (0.14)	1.51 (0.13)	5.24 (0.74)	6.85 (0.9)

*^a^Significant difference between The Mole Easy and The Mole Medium; ^b^significant difference between LightRace Easy and LightRace Medium; ^c^significant difference between The Mole Easy and LightRace Easy; ^d^significant difference between The Mole Medium and LightRace Medium.*

#### Step Characteristics

**FIGURE 2:**
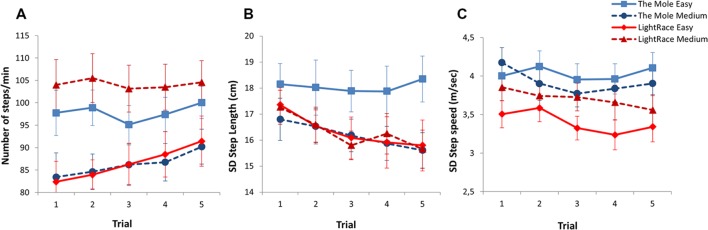
**Mean number of steps per minute (±SE) (A), SD Step Length (±SE) (B), and SD Step velocity (±SE) (C) for The Mole and LightRace in Easy and Medium level across five trials**.

As can be seen in **Table 2**, both Game and Level had an effect on number of steps per minute. Whereas participants took fewer steps per minute at the Medium compared to the Easy level when playing the Mole, the opposite was found for LightRace with participants taking more steps per minute at the Medium compared to the Easy level. For both games the difference between levels was significant (both *p*’s < 0.001), and there was a significant difference between the two exergames in both Easy (11.3, CI 95% 7.8 – 14.9, *p* < 0.001) and Medium (17.9, CI 95% 14.3 – 21.5, *p* < 0.001) levels. There was also an effect of Trial (*p* = 0.026), indicating that the number of steps taken increased from the first to the last trial in both games (see **Figure 2A**). In addition, there was an interaction between Game and Level [*F*(1,376) = 129.9, *p* < 0.001], indicating that the number of steps taken per minute increased from Easy to Medium level in LightRace, while it decreased from Easy to Medium when playing the Mole.

Game and Level also affected step length. Participants took longer steps on average when playing LightRace than when playing The Mole, and for both games, participants took longer steps at the Easy level than at the Medium level (both *p*’s < 0.001) (see **Table 2**). Furthermore, participants had more variation in step length at the Easy level than at the Medium level when playing The Mole (-1.9, CI 95% -2.4 – -1.0, *p* < .001), while there was no significant difference between the levels in LightRace (*p* = 0.935). For step length variation, there was an effect of Trial (*p* = 0.004), indicating that participants varied step length less the more times they played the same level and game (see **Figure 2B**). Furthermore, there was a significant interaction between Game and Level in step length variation [*F*(1,376) = 14.2, *p* < 0.001], indicating that participants had less variation in step length when playing the Medium level of the games, especially for The Mole.

Step velocity and variation in step velocity was affected by Game and Level as well. LightRace yielded higher step velocity, but less variation in step velocity in both Easy and Medium levels compared to The Mole (see **Table 2**). For The Mole, there was a significant difference between the two difficulty levels in step velocity (*p* = 0.005), but no significant difference in step velocity variation (*p* = 0.158), while LightRace displayed a significant difference between the levels in both step characteristics (both *p*’s < 0.001). Furthermore, there was a significant difference in step velocity between the two games at the Medium level (-1.5, CI 95% -1.8 to -1.1, *p* < 0.001), but no significant difference at the Easy level (*p* = 0.141). For variation in step velocity there was a significant difference between the games at both the Easy level (-0.6, CI 95% -0.8 to -0.5, *p* < 0.001) and at the Medium level (-0.2, CI 95% -0.4 to -0.1, *p* = 0.008). For variation in step velocity there was also an effect of Trial (*p* = 0.016), indicating that the variation in step velocity decreased overall from the first to the last trial (see **Figure 2C**). While participants increased their step velocity and variation in step velocity from Easy to Medium level when playing LightRace, both characteristics decreased in The Mole, yielding a significant interaction between Game and Level for both step velocity and step velocity variation [*F*(1,376) = 23.02, *p* < 0.001 and *F*(1,376) = 14.2, *p* < 0.001, respectively].

#### Upper Body Movements

Characteristics of upper body movements in AP- and ML-directions were also affected by Game and Level. As shown in **Table 2**, The Mole induced higher velocity and more variation in upper body velocity for both AP- and ML-directions compared to LightRace (all *p*’s < 0.001). For both games and both movement directions, playing the Easy level gave higher velocity and more variation in upper body velocity than at the Medium level (all *p*’s < 0.001). There were no Trial effects for any of the upper body movement characteristics. For upper body velocity in AP-direction, there was a significant interaction between Game and Level [*F*(1,376) = 29.9, *p* < 0.001], indicating that The Mole elicited higher velocity when taking forward and backward steps at the Easy level than at the Medium level, while there were no such difference in LightRace.

#### Movement Area and Movement Area Ratio

**FIGURE 3:**
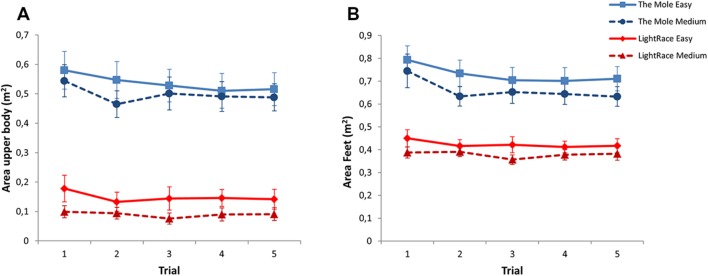
**Mean area (±SE) covered by the upper body (A) and mean area (±SE) covered by the feet (B) for The Mole and LightRace in Easy and Medium levels over five trials**.

Game and Level also affected the movement area covered by the feet and upper body, and their ratio. While playing the two games, the participants covered a larger area with both the upper body and the feet when playing The Mole compared to LightRace (see **Table 2**). In both The Mole and LightRace participants covered a larger area with both their feet and upper body when playing the Easy levels of the two games than the Medium levels (all *p*’s < 0.001) (see **Figures 3A,B**). For area covered by the feet, there was a significant Trial effect [*F*(1,377) = 5.4, *p* = 0.020], indicating that the participants decreased the area in which they moved their feet from the first to the last trial (see **Figure 2B**). There were no significant interactions for movement area in the upper body or the feet.

With respect to the movement area ratio, LightRace had a higher ratio than The Mole at both Easy (5.3 vs. 1.6) and Medium levels (6.9 vs. 1.5), indicating that participants to a larger extent moved their upper body with their feet in The Mole compared to LightRace. For The Mole, there was no difference between Easy and Medium levels (*p* = 0.867), while LightRace had a higher movement area ratio at Medium level than at the Easy level (1.5, CI 95% 0.8 – 2.2, *p* < 0.001). There was a significant interaction between Game and Level [*F*(1,376) = 10.37, *p* = 0.001], indicating that LightRace had an increase in the ratio from Easy to Medium level, while there was no such change in The Mole. There was no effect of Trial on movement area ratio.

## Discussion

The last decades have shown a rapidly growing interest to use exergames to increase physical activity and improve health and physical function in older adults (Laver et al., 2011; Verheijden Klompstra et al., 2014; Skjaeret et al., 2016). However, we lack in knowledge about how older adults actually move when playing these exergames, making it difficult to deduce whether the exergames prompt the exercise and functions intended, and interpret inconsistent findings from intervention studies. The current study is the first to study movement characteristics in detail while older adults played different stepping exergames, in order to investigate whether movement characteristics are influenced by the choice of game, difficulty level, and repeated trials.

We chose two stepping exergames for this study that came out positive in an earlier study (Skjaeret et al., 2015) and that share many similarities in terms of the technology used and the movements the players have to perform in order to play the game and score points. Nevertheless, our results indicate that stepping and upper body movements performed by older adults while playing the two exergames demonstrated important differences that can affect the intended exercise these games provide when used as an intervention.

Being able to vary stepping movements in terms of, e.g., step length and step direction is important in order for stepping and walking to be adaptive in daily life and to successfully achieve a task (Latash and Anson, 2006; Moe-Nilssen et al., 2010). The two exergames used in this study yielded different stepping characteristics with LightRace prompting the participants to take longer steps at a higher step velocity than when playing The Mole, but with less variation in both movement characteristics. This illustrates that when an intervention aims at improving variation in movement characteristics, the context of the games needs to be thoroughly examined prior to their use to ensure that players achieve this desired variation in step and upper body movements. With regards to step velocity, both games provided new targets as soon as the current target was hit, but in LightRace the players can get a bonus multiplier if they hit multiple targets in a row without any missteps. This bonus multiplier might have triggered the players to take faster steps in order to beat their own game score, hence increasing their step velocity. Thus, if velocity of stepping movement is in focus, having a trigger in the game that prompts the player to take new steps fast might be a solution.

Results in this study also showed that the characteristics of the game influence how large a movement area participants covered during playing. They had more upper body movements in both AP- and ML-directions as well as covering a larger movement area with both the feet and upper body when playing The Mole compared to LightRace. If the goal of an intervention is to induce stepping in a larger area and in different directions, evaluating game characteristics and game design might give an indication of how to make a choice. While game play in LightRace consists of stepping from a fixed circle in the middle of the game area, The Mole consists of a 3 × 3 grid where the targets appear randomly in the different areas, making the participants take steps in several different directions within a larger area. In addition, LightRace requires the player to step back into the middle of the circle before taking a new step, while for The Mole the player can take new steps in a different direction immediately after hitting the object on the screen. The game characteristics thus allow – and may even prompt - the player to take steps in several directions during game play.

Older adults have been found to be more unstable when shifting weight from double to single leg support compared to younger adults (Ihlen et al., 2012). This period of transfer or shifting of body weight has also been identified as the moment where most falls among older adults occur (Robinovitch et al., 2013). In the current study, the calculated ratio between feet and upper body movements served as an indirect proxy for weight shifts. This movement ratio turned out to be one of the largest differences between the two games used in this study. Players covered almost equal ground with their feet and upper body when playing The Mole, indicating that the upper body followed the movements of the feet to a large extent during each step. In contrast, in LightRace the upper body moved only a fifth or sixth of the step itself, indicating that the upper body was kept more stationary while the feet made tapping movements. These differences point to two separate but important aspects of balance exercises, depending on what the goals of the exercise are. Based on the results of the current study, if the focus of an exercise is on performing complete stepping movements that require transferring the body weight from one leg to the other, playing The Mole is the more appropriate choice. However, if the goal is to perform balance exercises by reducing the base of support, playing LightRace where participants stood on one foot while tapping the other on a target in front, behind or to the side, would be a preferred exercise. Both these abilities are important parts of exercises aiming to challenge balance in older adults (Sherrington et al., 2011) and should be taken into consideration when using exergames as exercise interventions for older adults.

Interestingly, this study not only showed a difference in the elicited movement characteristics between the two exergames, but also between the two difficulty levels within each game. By increasing the level of difficulty, almost all movement characteristics decreased, with the players in general taking shorter steps with less variation in length and velocity with a smaller movement area, especially for the upper body. However, there were some important differences between the two exergames as well.

When playing The Mole, participants had an overall decrease in movement from the Easy to the Medium level. This is likely linked to the additional cognitive challenge at the medium level of The Mole, which consists of a ladybug that one should avoid stepping on, as this would cost points. To avoid getting a reduction in points, some participants tried stepping over the ladybug, like one might naturally do outdoors. However, the game system was not sensitive to vertical motions and consequently, stepping over the ladybug was considered the same as stepping on it, costing the player points. This led some participants to decrease the size of their steps and make small, shuﬄing steps around the ladybug, while others stopped entirely, waiting for the ladybug to disappear so they could move straight toward the mole. Adding an additional cognitive element to an exergame is often perceived as fun and challenging by the players, as also shown in the present study, but might thus have unintended effects on the movements performed to play the exergame. This again illustrates the importance of knowing how games and choices within the games affect players’ movements if exergames are to be used to exercise specific functions. If the goal is to make the player perform and improve specific movement characteristics, the games and difficulty levels used to accomplish this needs to be chosen with care. In addition, the technology of the game system needs to be sensitive enough to ensure that the intended manner of playing the game is rewarded, for example by adding points to a game score, and that performing incorrect movements does not yield points. The competitive side of beating one’s previous score was in itself seen as a motivation to play the games by the participants. Therefore, it is important to ensure that participants perform the movement characteristics intended by the game when rewarding points.

In LightRace, an additional stepping direction was added at the Medium difficulty level, requiring the players to take steps backward as well as forward and sideways. One could expect that this would increase overall movements made by the participants. However, as the avatar on the screen was mirrored, several of the participants became confused and stepped in the wrong direction, especially for the backward/forward steps, leading to a reduction in stepping and upper body movements. Perhaps partly because of these initial errors, the total number of steps per minute and step velocity increased when playing the Medium difficulty level.

As this study illustrates, changing the game or the difficulty level changes players’ movement characteristics. Thus, if the goal of the exercise or intervention is to be able to carry out specific physical functions or specific aspects of balance, in other words, to perform specific movement characteristics, one cannot pick a game or difficulty level at random. It is important to select games with care based on the movement characteristics they elicit in the players while keeping the intended exercise in mind, and not only based the choice of game on aspects like convenience, promoted enjoyment of the game, the game technology and ease of use for the players. The same goes for designing and developing games for older adults. In recent years researchers have come up with several important aspects that should be taken into consideration when designing games for older adults (Ijsselsteijn et al., 2007; Gerling et al., 2012). However, little attention has been given to the movement characteristics of the players in the design process.

Another aspect that the current study illustrates is that repeated trials of the same game and same game level also can have an effect on the movement characteristics performed by the players. In other words, there might have been a potential learning effect occurring due to familiarization to the games. While the number of steps per minute increased from the first to the fifth trial, both step length variation, variation in step velocity, and area covered by the feet during game play decreased from the first to the last trial, illustrating that the participants were able to adapt their movements to the game requirements after playing an exergames a few times only. At the same time, the game score increased or remained the same, indicating that a reduction in movement characteristics did not affect the game score. If an exergame is to be used over a longer period of time, e.g., at home as a rehabilitation tool, being able to adapt movements during five attempts only might not ensure the intended exercise movements aimed for. Exergames have in the last decade been used in exercise and rehabilitation settings much due to their perceived enjoyment. However, little attention has been directed toward the potential learning effect that might affect movements negatively in the long run and make the games less fun to perform, resulting in low adherence to exercising. New technology provides great opportunities to save and store large amount of information from an exergaming session, which in turn can enable personalized feedback on how movements should be performed, as well as adjusting and adapting the games to each player.

The results of the current study illustrate that stepping and upper body movements performed by older adults when playing stepping exergames are influenced by the choice of game, how the difficulty level, and whether players play multiple trials, and it is therefore not indifferent which games are chosen to achieve specific aims. If the goal is to increase general physical activity, both exergames used in this study show promise as all participants took several steps with variation in step length, speed, and direction. However, if a rehabilitation goal is to train a specific function, one needs to evaluate different opportunities to find the best suited game that affect the movement characteristics in mind. In addition to finding the best suited game, game elements that appear or even disappear with increasing difficulty level should be taken into consideration. How will an additional cognitive load such as a ladybug in The Mole, or an additional movement direction such as in LightRace, affect the movement characteristics that are aimed for? In early stages of acquiring new movement skills, people often reduce their movements by freezing degrees of freedom, while later in the learning process releasing these degrees of freedom to allow for more flexible movements (Vereijken et al., 1992). In conventional forms of exercise as well, movements might decrease when adding an additional load or changing from one exercise to a new exercise. However, the change should not be so large that people are not able to achieve the desired movement characteristics at all.

There are some limitations to the current study that should be pointed out. Firstly, the present study compared only two different step-based exergames, but nevertheless found important differences in what movements were performed while playing them. In order to ensure that an appropriate game is chosen when aiming to exercise specific physical functions, in-depth analysis of movements performed during exergames is necessary, and this study is the first step in that direction. The current results illustrate that movement analysis during exergaming is necessary in order to understand the effect (or lack thereof) of exergaming. A second limitation is related to the number of trials. Although this study found some significant trial effects, five repetitions at each level in each game was not enough to further investigate the relationship between trials and the development of movement characteristics and game score. Future studies should include more game trials over a longer period in order to investigate the relationship between the game score and the movements made by the participants, and to ensure that the intended movements are maintained over extended playing time. Future studies should also include testing of cognitive function even in a relatively healthy sample of older adults. This would allow investigating the relationship between cognitive function and changes in gameplay at harder levels. Another limitation is that the steps were identified by velocity of the toe markers only, whereas most step detection algorithms make use of both toe and heel information. As the Oqus motion capture cameras could not be placed behind the participants because of interference with the gaming cameras, the heel markers were often occluded from view, making them unreliable to use in the step detection algorithms. For the purpose of the current study, this was less relevant as we compared across games, levels, and trials, but when accurate details of individual steps need to be detected, algorithms should be based on both heel and toe markers to ensure the most accurate identification of step characteristics.

## Conclusion

The current study provides several important insights regarding the use of exergames by analyzing the movements performed by older adults when playing step-based exergames. Although exergames in general seem effective in increasing general physical activity among older adults, there has been no focus on whether stepping exergames train specific movement characteristics important for maintaining balance and gait in real life, such as weight shifting and variation in step length, speed, and direction. The results from the current study illustrate that it is not irrelevant which games are chosen to exercise these functions and show that movement characteristics are affected by the game, level of difficulty, and (even short-term) experience with the games. For future use of exergames in the context of exercise or rehabilitation at home, it is important to select exergames with care, taking into account what movement characteristics one wants the player to perform, how to manipulate difficulty level to maintain motivation without sacrificing the quality of the movements, and how to use the scoring of points to ensure that the proper movements are executed.

## Author Contributions

NS-M, EV, and BV designed the study and collected the data. NS-M, EV, EI, X-CT, and BV contributed to data analysis and interpretation. NS-M drafted the manuscript, and EI, JH, and BV provided critical comments and contributed to revisions. All authors approved the final version of the manuscript for submission.

## Conflict of Interest Statement

The authors declare that the research was conducted in the absence of any commercial or financial relationships that could be construed as a potential conflict of interest.
